# CT imaging of malignant peritoneal mesothelioma: A case report

**DOI:** 10.1016/j.radcr.2022.01.021

**Published:** 2022-02-08

**Authors:** Agnes Triana Basja, M. Hidayat Surya Atmaja

**Affiliations:** a Radiology Resident, Department of Radiology, Faculty of Medicine Universitas Airlangga, Dr. Soetomo General Academic Hospital, Surabaya, Indonesia; b Abdominal Radiologist, Department of Radiology, Faculty of Medicine Universitas Airlangga, Dr. Soetomo General Academic Hospital, Surabaya, Indonesia

**Keywords:** Mesothelioma, Peritoneal

## Abstract

The peritoneal cavity is the second most common location for mesothelioma with only 10.5% of 10,589 mesothelioma cases reported in the United States between 1973 until 2005 and known with poor prognosis. Diagnosing malignant peritoneal mesothelioma is a challenge for many clinicians because the symptoms are often non-specific. Therefore, the emergence of computer tomography (CT) features knowledge and another finding is an essential aspect in establishing the diagnosis. We present a case 43-years-old man with no history of malignancy presented with abdominal distention, weight loss, and decreased appetite for 2 months. To pursue the proper diagnosis an abdominal CT was performed and revealed diffuse, irregular thickening of the parietal peritoneum with multiple nodules, and the chest CT revealed a left-sided pleural effusion with multiple nodules in both lungs. Concomitant with the imaging result, histopathological examination and immunohistochemical analysis of a core biopsy of the peritoneal nodule showed a malignant round cell tumor indicating the source of the mesothelioma.

## Introduction

It has been reported that only 10.5% of 10,589 mesothelioma cases reported in the United States between 1973 and 2005 originated from the peritoneum; moreover, in industrialized countries, the occurrence rate of this disease ranges from 0.5-3.0 cases per million in men, and from 0.2-2.0 cases per million in women [1]. Peritoneal mesothelioma occurs in all age groups, however most cases occur in the fifth and sixth decades [2]. It is widely accepted that mesothelioma is related to exposure to toxins through industrial pollutants, notably asbestos [3]. Mesothelioma can also occur without direct asbestos exposure and can be associated with silica and radiation [4]. Moreover, the clinical features of malignant peritoneal mesothelioma (MPM) are non-specific; in most cases the patients report abdominal distension, pain, nausea, anorexia, and weight loss [1]. According to the literature, there are 3 main patterns of clinical presentation: abdominal pain, abdominal distension, and a combined presentation. These symptoms are correlated with computer tomography (CT) imaging manifestations. In CT imaging, mesothelioma cases are assigned 3 additional characteristics: dry type, wet type, and mixed type [2]. CT imaging of MPM resembles peritoneal carcinomatosis [5]. Lastly, an examination of anatomical pathology and immunohistochemistry must be carried out to establish the diagnosis of MPM [2].

## Case report

**Fig. 1:**
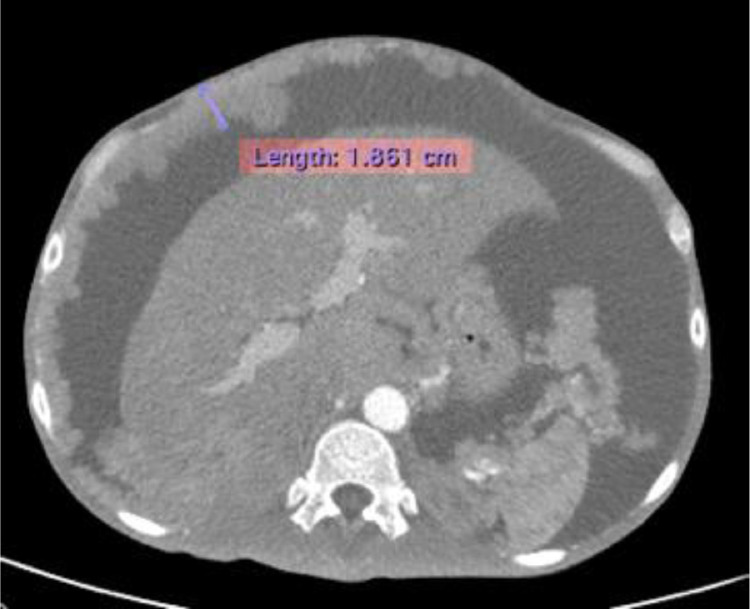
Axial view showed diffuse, irregular thickening of the parietal peritoneum up to 1.8 cm in thickness.

**Fig. 2:**
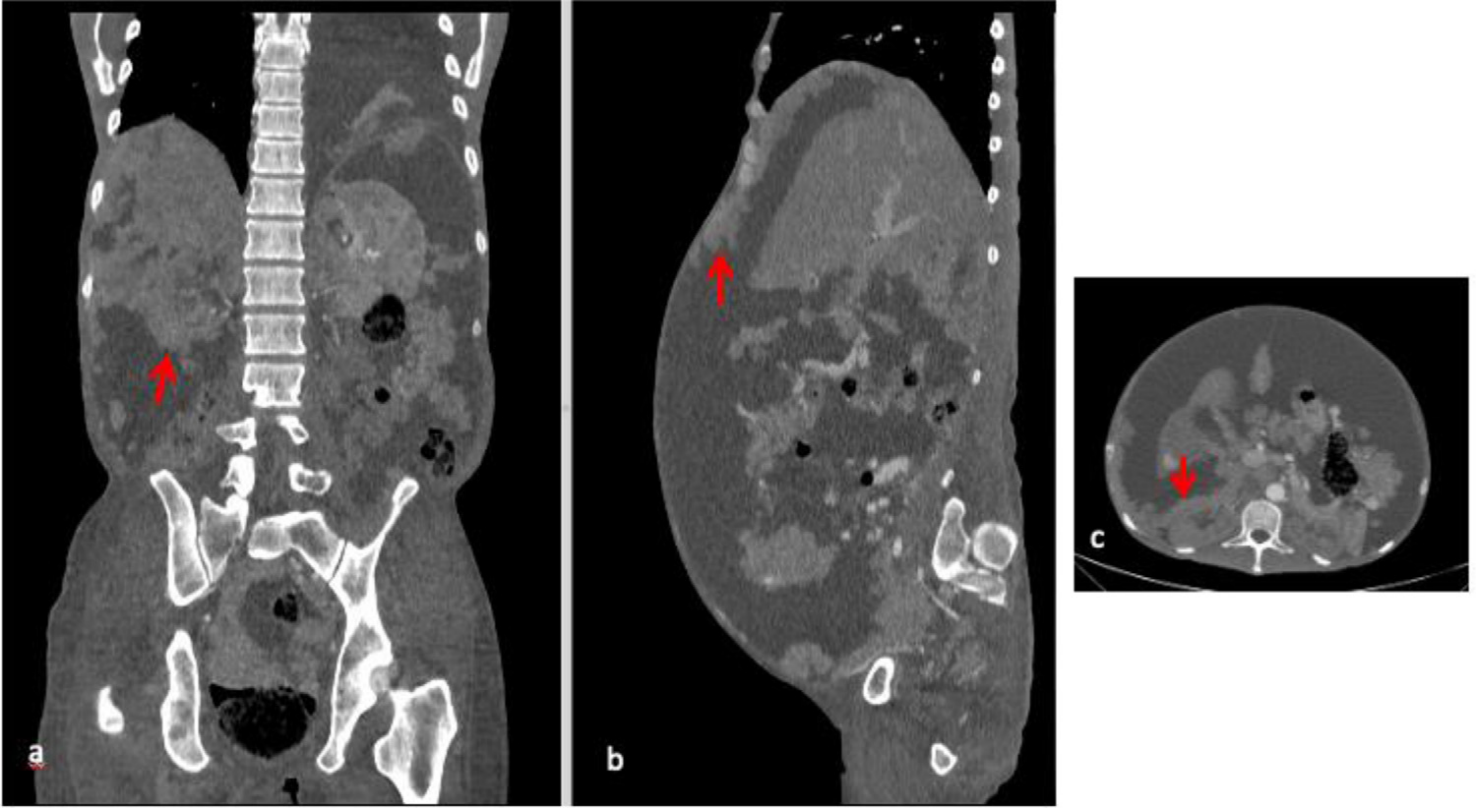
(A) Coronal and (C) axial views showed multiple nodules (red arrows) on the visceral peritoneum of the kidney. (B) Sagittal view showed multiple nodules (red arrow) on the parietal peritoneum.

**Fig. 3:**
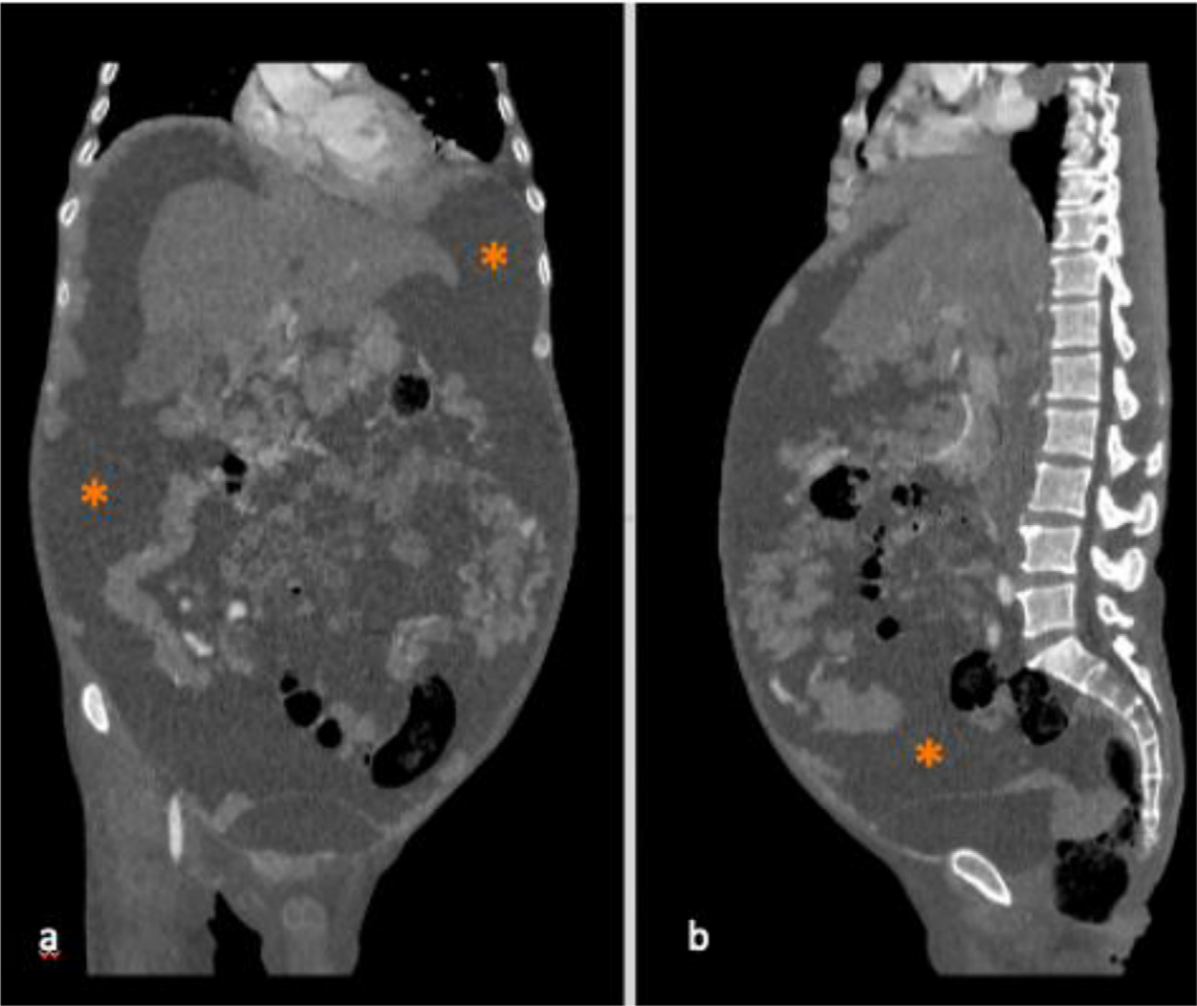
(A) Coronal view and (B) sagittal view showed large volume ascites (asterisks) in both the abdominal cavity and pelvic cavity.

**Fig. 4:**
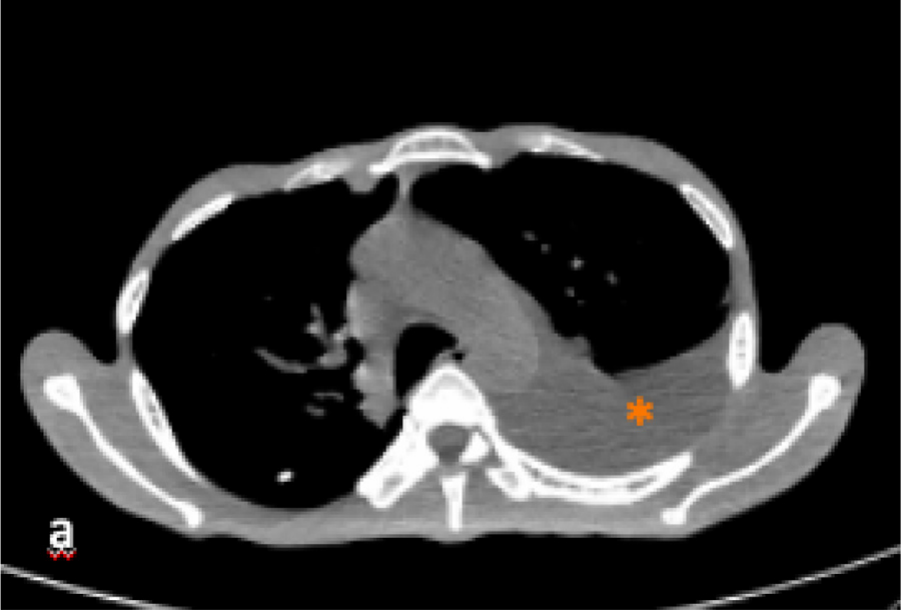
(A) Axial view with mediastinal window showed left-sided pleural effusion (asterisk).

**Fig. 5:**
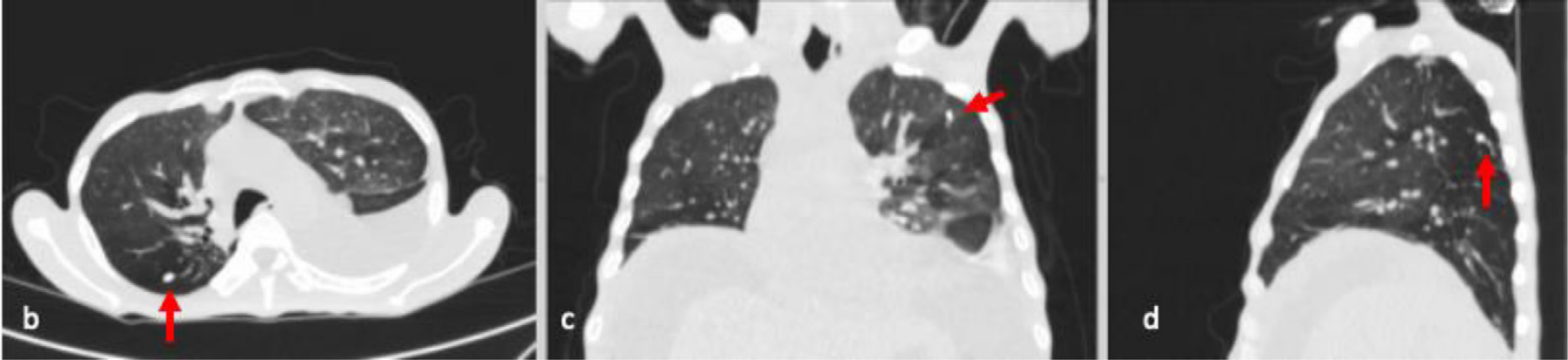
(A) Axial, (B) coronal, and (C) sagittal views showed multiple nodules (red arrows) in both lungs.

**Fig. 6:**
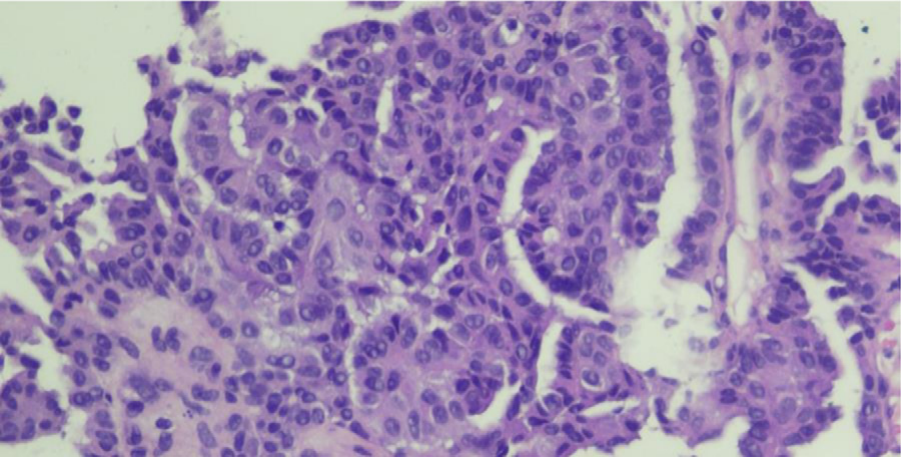
Peritoneal mesothelioma: an epithelioid component with eosinophilic cytoplasm.

A 43-years-old man with no history of malignancy presented with abdominal distention, weight loss, and decreased appetite for 2 months. A physical examination was carried out and ascites permagna was noted. The tumor markers, serum alpha-fetoprotein, CA-125, and CA-19.9 were within normal limits. Abdominal CT revealed diffuse, irregular thickening of the parietal peritoneum with multiple nodules. The thickening was up to 1.8 cm on visceral peritoneal surfaces (Figs. 1 and 2). A large volume of ascitic fluid was found in the abdominal cavity and pelvic cavity (Fig. 3). Chest CT revealed a left-sided pleural effusion with multiple nodules in both lungs (Figs 4 and 5). Following histopathological examination and immunohistochemical analysis of a core biopsy taken from a peritoneal nodule, a malignant round cell tumor was discovered to be the source of the mesothelioma (Fig. 6).

## Discussion

Malignant peritoneal mesothelioma (MPM) is the second most common type of mesothelioma; it accounts for around 30% of all cases of malignant mesothelioma, with an overall incidence of 2-2.6 cases per million per year. The development of peritoneal mesothelioma is associated with a history of asbestos exposure; some patients have no history of asbestos exposure, as in this case. According to the literature, exposure to mica, talc, thorium, and simian virus 40 (SV40) infection could be a risk factor for mesothelioma [6].

Clinical features of MPM are non-specific in early stages and thus most cases are diagnosed at an advanced stage. The clinical presentation of the tumor's spread within the abdominal cavity may vary and is associated with the accumulation of ascites and the presence of mass growth leading to the primary symptom of abdominal distension. MPM is generally divided into 2 types: diffuse and local. Most patients exhibit the diffuse peritoneal type. Those with diffuse malignant peritoneal mesothelioma (DMPM) usually present with abdominal pain or distension [5]. Moreover, in 30%-80% of patients, abdominal pain is the second most common symptom, occurring in around 27%-58% of cases [7]. Patients might report non-specific symptoms such as early satiety, anorexia, weight loss, vomiting, constipation, and/or diarrhea. On the other hand, 8% of the patients are diagnosed unexpectedly [7]. In this case, the patient had complained of abdominal distention, weight loss, and decreased appetite.

In general, according to Sudhantiram et al. (2012), CT images of mesothelioma cases are categorized into 3 types:

• Dry type: the presence of a large peritoneal mass or multiple, diffuse, small peritoneal nodules with little or no ascites.
• Wet type: a predominant presence of ascites with or without multiple nodules or small plaques.
• Mixed type: the combination of dry and wet type [6].

MPM has several manifestations on CT: thickening of the peritoneum, mesentery, and pleura; multiple nodules of various sizes and shapes; the presence of mesenteric or omental infiltration; ascites; adenopathy; and bone destruction.

### 1. Irregular wall thickening

Irregular peritoneal thickening of >1 mm was found in some MPM patients [8].

### 2. Multiple nodules

Tumor growths may spread to the parietal and visceral peritoneum of intra-abdominal organs. MPM presents with involvement of the omentum, diaphragm, liver, small and large intestines, and mesentery [1]. Thus, one of the hallmarks of MPM is indicated by the presence of scattered nodules on the surface of the parietal and visceral pleura [9].

### 3. Ascites

Ascites is radiologically defined as the presence of high fluid density (>20 HU) or low fluid density (<10 HU).

In cases where ascites has spread through the entire abdominal cavity and to pelvic cavity, it is referred as extensive ascites. In moderate ascites, fluid is localized around the liver and spleen. In mild ascites, there is only a small amount of fluid present [8].

### 4. Pleural effusion

In some patients with MPM, pleural manifestations are commonly found as pleural calcifications and recurrent pleural effusions [1].

In this case, multiple nodules of up to 1.8 cm in thickness on the parietal and visceral peritoneal surfaces were visualized using abdominal CT, which also revealed the mixed type with diffuse, irregular thickening. A large volume of ascitic fluid was present in the abdominal cavity extending to the pelvic cavity. Furthermore, chest CT revealed a left-sided pleural effusion with multiple nodules in both lungs. The CT images demonstrated features of MPM, though they could have been features of peritoneal carcinomatosis as well. Nevertheless, the immunohistochemical examination revealed a mesothelioma of the peritoneum.

The recommended treatment since the 1990s is cytoreduction combined with hyperthermic intraperitoneal chemotherapy (HIPEC) which has been considered in patients with peritoneal mesothelioma. This treatment is a promising treatment for patients with long-term survival of 5 years increasing to 50% in these patients. Despite these advances in treatment, globally most patients with MPM receive palliative care or systemic chemotherapy. This incident has been noted in previous studies, in which most patients were treated with palliative therapy or diagnosed during autopsy [10]. This patient, currently intraperitoneal chemotherapy is in progress after being diagnosed.

## Conclusion

Malignant peritoneal mesothelioma (MPM) is a rare occurrence; despite this, these diagnoses could easily lead to misdiagnosis. CT images can diagnose and categorize the type of MPM, even though the gold standard is through immunohistochemical examination. In addition, the awareness and knowledge of imaging and other investigations is therefore essential in establishing the diagnosis.

## Consent and Ethic Comitee Approval

Written consent has been obtained from the patient as there is no patient identifiable data included in this case report. This study has met the ethical principle and already got approval from Research Ethics Committee from Dr. Soetomo General Hospital, Surabaya.

## Patient Consent

Written informed consent was obtained from the patient for the publication of this case report.

## Funding

None
